# The influence of demography and local mating environment on sex ratios in a wind-pollinated dioecious plant

**DOI:** 10.1002/ece3.465

**Published:** 2013-02-06

**Authors:** Melinda Pickup, Spencer C H Barrett

**Affiliations:** Department of Ecology and Evolutionary Biology, University of Toronto25 Willcocks Street, Toronto, ON, Canada, M5S 3B2

**Keywords:** dioecy, female-biased sex ratios, gametophytic competition, local mating environment, plant density, sex ratios, wind pollination

## Abstract

Negative frequency-dependent selection should result in equal sex ratios in large populations of dioecious flowering plants, but deviations from equality are commonly reported. A variety of ecological and genetic factors can explain biased sex ratios, although the mechanisms involved are not well understood. Most dioecious species are long-lived and/or clonal complicating efforts to identify stages during the life cycle when biases develop. We investigated the demographic correlates of sex-ratio variation in two chromosome races of *Rumex hastatulus*, an annual, wind-pollinated colonizer of open habitats from the southern USA. We examined sex ratios in 46 populations and evaluated the hypothesis that the proximity of males in the local mating environment, through its influence on gametophytic selection, is the primary cause of female-biased sex ratios. Female-biased sex ratios characterized most populations of *R.* *hastatulus* (mean sex ratio = 0.62), with significant female bias in 89% of populations. Large, high-density populations had the highest proportion of females, whereas smaller, low-density populations had sex ratios closer to equality. Progeny sex ratios were more female biased when males were in closer proximity to females, a result consistent with the gametophytic selection hypothesis. Our results suggest that interactions between demographic and genetic factors are probably the main cause of female-biased sex ratios in *R. hastatulus*. The annual life cycle of this species may limit the scope for selection against males and may account for the weaker degree of bias in comparison with perennial *Rumex* species.

## Introduction

Sex ratios in equilibrium populations of dioecious organisms are expected to be close to equality (1:1 ratio of females and males) as a result of negative frequency-dependent selection (Fisher 1930; Edwards 2000; Hardy 2002). However, surveys of the sex ratio in dioecious plants commonly report significant deviations from equality (Delph 1999; Barrett et al. 2010; Sinclair et al. 2012). A recent compilation of plant sex ratios (Fieldet al. 2012a) in 243 species of flowering plants representing 123 genera and 61 families found significantly biased ratios in half (50.2%) of all species, with male bias (31.3%) nearly twice as common as female bias (18.9%). The frequent occurrence of biased sex ratios in plant populations raises the question of what ecological and genetic factors might cause deviations from equality and when during the life cycle these become evident.

Determining the mechanisms causing biased sex ratios in plants is more difficult than in many animal groups because of their more complex life histories and demography. Dioecious plants are most commonly perennial and many are long-lived and either clonal or woody (Renner and Ricklefs 1995). As a result, most data on sex ratios are based on flowering plants, because the sexual identity of nonreproductive individuals cannot be determined without sex-specific genetic markers (e.g. Eppley et al. 1998; Stehlik and Barrett 2005; Shelton 2010). In clonal species, sex ratios are usually determined from surveys of flowering ramets and the extent to which ramet sex ratios reflect genet sex ratios is usually not known (Barrett and Thomson 1982). Moreover, many dioecious populations, especially those with extensive clonal propagation, may be in a nonequilibrium state when sampled. Under these circumstances, sex ratios could be influenced by founder events and progress to sex-ratio equilibrium may often be quite protracted, especially where sexual recruitment is infrequent (Barrett et al. 2010). These difficulties complicate studies of the causes of sex-ratio bias in many dioecious plants, but are reduced in annual species because the sex of individuals and genet sex ratios are more easily determined. Unfortunately, dioecy is relatively uncommon in annual plants limiting the choice of study organism.

Since the pioneering work of Correns on *Rumex* (Correns 1922), studies of sex-ratio variation in this genus have indicated a relation between biased sex ratios and pollination intensity, here defined as the number or “load” of pollen grains deposited on stigmas. Subsequent pollination studies of *Rumex* species have confirmed Correns' original finding that larger pollen loads are associated with female-biased sex ratios (Rychlewski and Zarzycki 1975; Conn and Blum 1981; Stehlik and Barrett 2006; Field et al. 2012b). The differential performance of female- versus male-determining microgametophytes resulting in selective fertilization, a phenomenon known as certation (Correns 1922), appears to best explain these results. Because the flowers of *Rumex* species posses a single ovule, gametophytic competition is likely to be particularly sensitive to pollination intensity. For example, garden experiments (Stehlik and Barrett 2006) and studies of natural populations (Stehlik et al. 2008) of *R. nivalis* indicate that females in close proximity to males capture more pollen and produce more female-biased progeny sex ratios in comparison with more isolated females. In this long-lived alpine perennial, considerable gender-based mortality of vegetative plants also contributes toward the female-biased sex ratios that characterize natural populations (mean 0.82, range 0.69–0.85, *n* = 18 populations; Stehlik and Barrett 2005). These findings imply that progeny sex ratios are likely to be affected by the demographic characteristics of populations, particularly, the local density of plants and their sexual identity.

Species of *Rumex* with female-biased sex ratios possess heteromorphic sex chromosomes (females XX, males XY or XY_1_Y_2_; Smith 1963; Vyskot and Hobza 2004). This association between sex chromosomes and female bias is not restricted to *Rumex*, but has been found in other flowering plant families with sex chromosomes (Field et al. 2012a). The occurrence of this association has led to the hypothesis that the poor performance of male gametophytes may be associated with the degeneration of nonrecombining portions of Y-chromosomes (Smith 1963; Lloyd 1974). This could lead to the reduced efficacy of purifying selection and the accumulation of deleterious mutations with subsequent fitness effects on the viability of both gametophytes and sporophytes (Lloyd 1974; Charlesworth and Charlesworth 2000; Charlesworth 2002; Stehlik and Barrett 2005).

Here, we investigate ecological and genetic factors influencing sex-ratio variation in *R. hastatulus*. Specifically, the goals of our study were to: (1) examine deviations from equality and among-population heterogeneity in sex ratios for populations of *R. hastatulus* and determine if population characteristics (size, density, and the proportion of nonreproductive plants) can explain among-population variation in sex ratios; (2) assess sex-ratio variation within populations to examine if spatial variation in the density of *R. hastatulus* plants was associated with local variation in sex ratios; (3) investigate whether the local mating environment influences progeny sex ratios by determining the effect of male proximity on progeny sex ratios for focal females. Following the certation hypothesis, we predicted that females in close proximity to males would produce more female-biased sex ratios than those that were more isolated; and (4) using progeny arrays from similar mating environments examine maternal variation in sex ratios.

## Methods

### Study species and population sampling

*Rumex hastatulus* (Polygonaceae) is a wind-pollinated, annual (rarely short-lived perennial) dioecious weed that is distributed across the southern regions of the USA from Texas to North Carolina and Florida. It is a colonizer of disturbed areas and is usually found on sandy, well-drained soils. It is a cytologically complex species, including two main geographically widespread chromosome races (Smith 1963), the North Carolina karyotype (females = XX, 2*n* = 8; males = XY_1_Y_2_; 2n = 9), and the Texas karyotype (females XX, males XY, 2n = 10). Populations of the Texas race are distributed across four states; Texas (TX), Oklahoma (OK), Arkansas (AK), and Louisiana (LA), whereas populations of the North Carolina race occur in North Carolina (NC), South Carolina (SC), Georgia (GA), Alabama (AL), and Florida (FL). The species is a winter annual, with plants overwintering as a basal rosette until spring when they produce one to several flowering stems.

We measured sex ratio, population size, and plant density in 46 populations of *R. hastatulus* from the southern USA. The populations span the geographic range of the North Carolina and Texas chromosome races and encompass the observed variation in population size and plant density within each chromosome race. We surveyed 23 populations each from the North Carolina and Texas races (see Table 1) over a 5-week period in May and June 2009, which represented a range of population sizes (TX race range = 66–≍2,000,000, NC race range = 10–≍556,000) and plant densities (TX race range = 0.21–122.4 plants m^−2^, NC race range = 0.04–34.3 plants m^−2^). Direct counts were used to obtain the sex ratio (females/females + males) and total population size (*n* = females + males) for the 16 populations with <2000 individuals. In these populations, we estimated plant density by dividing the total number of plants by the area of the population (m^2^). For large populations (≥2000 plants, *n* = 30), we estimated sex ratio by counting the number of females and males in 6–36 (median = 15.5) quadrats stratified along each of four randomly positioned transects (total quadrat *n* for each population = 24–113, median = 60.5). Due to differences in population size (and thereby area), transect length varied for each population sampled. To account for among-population variation in average density (mean density = 21.7 plants m^−2^, range = 0.08–122.4 plants m^−2^), and to ensure a minimum sample size of 450 plants per population, we used either 0.5, 1 or 2 m^2^ quadrats. We calculated plant density in these populations by dividing the number of plants (females + males) by the total quadrat sampling area. Within populations, we estimated local plant density (plants m^−2^) for each quadrat to examine the relation between local density and sex-ratio variation (described below). We sampled an average of 995 plants in each population (range = 10–4251, see Table 1). Sex could be determined from buds, flowers, or by the presence or absence of fruits, for all sampled individuals in the 23 Texas race populations. For the North Carolina race, nonreproductive plants were present in 19 populations and ranged in frequency from 0.1% to 32.0% of the populations sampled (Table 1).

**Table 1:** Sex ratio, location, plant density (plants m^−2^), and size (pop. size) of the 46 surveyed populations of *Rumex hastatulus*

Race	Pop.	Location	Latitude	Longitude	Density	Pop. size	*n*	F	M	NR	% NR	Sex ratio	*G*
NC	GA-GLA	Gladys, GA	31°28′55″	83°14′16″	5.61	131333	899	548	350	1	0.1	0.61	44.0***
NC	GA-BEL	Belfast, GA	31°50′35″	81°17′3″	5.30	933	933	468	394	71	7.6	0.54	6.4**
NC	GA-STA	Statesboro, GA	32°27′9″	81°50′55″	34.30	13789	686	419	267	0	0.0	0.61	34.0***
NC	FL-JAS	Jasper, FL	30°34′3″	83°4′31″	1.30	761	761	460	236	45	5.9	0.66	73.4***
NC	FL-GAI	Gainsville, FL	29°41′25″	82°26′24″	12.06	76898	579	331	204	30	5.2	0.62	30.4***
NC	FL-MIC	Micanopy, FL	29°30′44″	82°13′60″	0.04	18	18	12	6	0	0.0	0.67	2.0^NS^
NC	FL-HAM	Hammock, FL	29°4′5″	82°38′48″	5.10	166	166	117	49	0	0.0	0.70	28.7***
NC	FL-CED	Cedar Keys, FL	29°14′12″	82°56′13″	0.78	692	692	351	205	136	19.7	0.63	38.8***
NC	FL-CHI	Chiefland, FL	29°31′44″	82°53′4″	6.24	556340	1772	382	300	80	4.5	0.56	9.9***
NC	FL-MAR	Mariana, FL	30°48′43″	85°11′28″	10.24	115451	983	566	369	48	4.9	0.61	41.8***
NC	AL-BRU	Brundidge, AL	31°43′60″	85°50′25″	13.10	28800	576	365	200	11	1.9	0.65	48.9***
NC	AL-GRE	Greenville, AL	31°50′2″	86°43′9″	2.20	895	895	524	365	7	0.8	0.59	28.6***
NC	AL-BEL	Belleville, AL	31°23′33″	87°7′13″	2.96	24	24	12	12	0	0.0	0.50	0.0^NS^
NC	AL-BRE	Brewton, AL	31°4′58″	86°59′56″	10.90	77403	741	476	245	20	2.7	0.66	75.3***
NC	SC-PRO	Prosperity, SC	34°6′29″	81°26′14″	0.40	827	827	497	295	35	4.2	0.63	52.1***
NC	SC-MAR	Marion, SC	34°10′58″	79°29′13″	31.03	348876	879	368	230	281	32.0	0.62	32.1***
NC	SC-BRA	Branchville, SC	33°15′3″	80°48′27″	19.85	8738	1249	477	228	272	21.8	0.68	89.9***
NC	GA-ELA	Ellaville, GA	32°15′6″	84°16′47″	0.69	2757	797	503	250	44	5.5	0.67	86.7***
NC	NC-ELI	Elizabethtown, NC	34°38′21″	78°46′24″	1.54	311	304	186	102	16	5.3	0.65	24.9***
NC	NC-ROS	Roseboro, NC	34°58′8″	78°32′56″	3.17	17086	493	256	129	61	12.4	0.66	42.7***
NC	NC-HIC	Hickory, NC	36°6′59″	77°48′35″	4.03	17446	452	265	154	31	6.9	0.63	29.8***
NC	NC-BAT	Bath, NC	35°31′32″	76°52′18″	5.90	41173	573	364	187	22	3.8	0.66	57.9***
NC	NC-KIN	Kinston, NC	35°15′15″	77°36′3″	0.08	10	10	6	3	1	10.0	0.67	1.0^NS^
TX	LA-MAN	Many, LA	31°27′41″	93°39′39″	27.15	7466	577	348	229	0	0	0.60	24.7***
TX	LA-BEN	Benson, LA	31°51′1″	93°41′18″	2.79	6027	492	308	184	0	0	0.63	31.6***
TX	LA-DER	De Ridder, LA	30°53′39″	93°18′51″	4.25	1115	1115	665	450	0	0	0.60	41.7***
TX	TX-BUC	Buckhorn, TX	30°45′41″	93°40′33″	88.56	1448221	4251	2800	1451	0	0	0.66	435.6***
TX	TX-WEL	Wellborn, TX	30°33′27″	96°20′9″	3.82	457	457	275	182	0	0	0.60	19.1***
TX	TX-LIV	Livingston, TX	30°41′58″	94°47′59″	65.95	73732	1319	820	499	0	0	0.62	78.9***
TX	TX-COL	College Station, TX	30°35′36″	96°18′31″	0.21	153	153	108	45	0	0	0.71	26.7***
TX	TX-GID	Giddings, TX	30°8′19″	96°55′9″	14.34	32800	1377	880	497	0	0	0.64	107.9***
TX	TX-ROS	Rosebud, TX	31°7′3″	96°51′37″	81.10	1978464	1622	1051	571	0	0	0.65	144.2***
TX	TX-GRO	Groesbeck, TX	31°30′49″	96°28′50″	29.60	75480	1184	783	401	0	0	0.66	125.5***
TX	TX-BUF	Buffalo, TX	31°26′10″	96°2′0″	103.80	78109	1246	790	456	0	0	0.63	90.6***
TX	TX-CRO	Crockett, TX	31°20′7″	95° 27′ 58″	4.80	441	441	302	139	0	0	0.68	61.7***
TX	TX-MTE	Mt Enterprise, TX	31° 56′ 19″	94°42′25″	1.40	1188	1188	764	422	0	0	0.64	100.0***
TX	TX-ATH	Athens, TX	32°11′5″	95°48′12″	21.90	1149421	1752	955	799	0	0	0.54	13.9***
TX	TX-MTP	Mt Pleasant, TX	33°10′28″	94°59′17″	122.40	722649	2908	1716	1191	0	0	0.59	95.3***
TX	OK-WIL	Willis, OK	33°53′48″	96°50′7″	10.70	5392	514	261	253	0	0	0.51	0.1^NS^
TX	OK-ROF	Roff, OK	34°41′3″	96°45′44″	1.44	4098	1358	795	563	0	0	0.59	39.8***
TX	OK-RAT	Rattan, OK	34°9′27″	95°24′48″	44.20	89505	884	569	315	0	0	0.64	74.0***
TX	OK-BAC	Bache, OK	34°53′36″	95°37′55″	60.60	756014	3335	2005	1400	0	0	0.59	108.1***
TX	TX-KEN	Kennard, TX	31°19′31″	95°22′12″	16.28	330962	1303	711	593	0	0	0.55	10.7**
TX	TX-SUL	Sulphur Springs, TX	33°5′46″	95°38′51″		66	66	37	29	0	0	0.56	1.0^NS^
TX	TX-WES	Wesley Chapel, TX	31°21′18″	95°31′25″	52.10	1027985	2084	1365	719	0	0	0.65	203.6***
TX	TX-OAK	Oakwood, TX	31°33′39″	95°54′6″	41.05	1090534	821	483	338	0	0	0.59	25.7***
												***G***_**H**_	293.1***

*n* = total sample size, F = number of females, M = number of males, NR = Number of nonreproductive plants, and% NR = nonreproductive plants as a percent of the total sample. Sex ratio is the number of females (F) as a proportion of the total sample (*n*). *G*-statistics (*G*) and *G*-heterogeneity test statistics (*G*_H_) are indicated with the level of significance.

*** *P* < 0.001.

** *P* < 0.01.

NS, *P* > 0.05.

### Maternal mating environment

To investigate whether male proximity was associated with female-biased sex ratios, we examined offspring sex ratios from focal females from a range of mating environments (low to high male density) in four populations of the Texas race. We used variation in male proximity as a proxy for variation in pollen load size, as this has been demonstrated in populations of other *Rumex* species (e.g. Stehlik et al. 2008) and for experimental arrays of *R. hastatulus* (M. Pickup, D. L. Field and S. C. H. Barrett unpublished data). The four populations were: Rosebud (TX-ROS), Oakland (TX-OAK), Kennard (TX-KEN), and Athens (TX-ATH). The populations were chosen because they contained considerable local variation in plant density that enabled us to locate focal females in high (distance to the first 10 males (*d*_10_) <40 cm radius), medium (40 ≤ *d*_10_ ≤ 120 cm radius), and low (*d*_10_ >120 cm radius) density patches. In each population, we haphazardly sampled 24–27 females; hence, they were evenly distributed across the three density treatments. For each focal female, we measured the distance to the nearest 10 males (*d*_10_). For the focal female and each of the 10 males, we measured plant height from the soil surface to the tip of the longest stem and counted the number of flowering stems and number of inflorescences. For each focal female sampled, we collected open-pollinated seed families by bulking seed from three inflorescences.

To determine progeny sex ratios for the four populations, we germinated 29–60 seed from each focal female during 2010. Seeds were germinated in petri dishes on moist filter paper in a growth cabinet maintained at 20°C for 12 h and 10°C for 12 h with continuous light. For all populations, seedlings were individually transplanted after ∼14 days to 5-cm pots containing Pro-Mix BX (peat moss, vermiculite and perlite) and NPK fertilizer (20:20:20) and these were grown to flowering in a glasshouse at 20–24°C. Average germination and range for families within each populations were: TX-OAK, mean = 87.6%, range = 63.0–97.0%; TX-ROS, mean = 88.6%, range = 61.1–100%; TX-ATH, mean = 80.6%, range 40.0–100%; TX-KEN, mean = 96.5%, range 93.0–100%). For all populations, plants from each focal female were monitored every second day for 12 weeks and the sex of each plant was determined on flowering. Survival from germination to flowering was high in all populations (96.9–99.5%). The total sample size for flowering individuals in the four populations were TX-OAK *n* = 1233, TX-ROS *n* = 1324, TX-ATH *n* = 732, TX-KEN *n* = 788. We used a larger sample of progeny per focal female in TX-OAK and TX-ROS to enable examination of among-family heterogeneity in sex ratios within each mating environment.

## Statistical analysis

### Sex-ratio bias and among-population variation in sex ratios

We used a goodness-of-fit test (*G*-Test) in R (R Core Development Team 2008) to examine if the sex ratio of each of the 46 populations of *R. hastatulus* was significantly different from the expectation of 0.5 (equal number of females and males). We then used heterogeneity *G*-tests (Sokal and Rohlf 1995) using a script written in R to assess if there was significant among-population heterogeneity in sex ratios. We used a Generalized Linear Model (GLM) (logistic regression) with a binomial distribution and a logit link function to examine if there was a significant difference in sex ratio between the chromosome races. Generalized Linear Model (GLM, logistic regression) analysis was also used to examine if population size and population density contribute toward among-population variation in sex ratio. For this model, population size and density and their interaction were fitted sequentially in the model. One population (TX-SUL) was omitted from this analysis due to the absence of a population density estimate. We also used a GLM (logistic regression) to determine if the proportion of nonreproductive individuals in the population sample was associated with variation in sex ratios for the 19 populations from the NC race in which vegetative plants were present. These, and subsequent GLM analyses, were undertaken in Genstat for Windows 13th Edition; VSN International, Oxford UK.

### Local density and within-population variation in sex ratio

For 22 populations of *R. hastatulus* where sex ratio was sampled using quadrats stratified along random transects, we used a Generalized Linear Mixed Model (GLMM, logistic regression) with a binomial distribution and a logit link function to examine if there was a relation between the density of *R. hastatulus* and local sex ratio within populations. For this analysis, population and density (and their interaction) were added to the fitted model and transect and quadrat (nested within transect) added to the random model. Each population was then analyzed independently due to the significant interaction between population and density (see Results). For these models, density was added as the fixed term in the model and transect (and quadrat nested within transect) fitted to the random model.

### Maternal mating environment (male proximity) and among-family heterogeneity in sex ratios

To investigate the relation between progeny sex ratio and the mating environment of each focal female, we used GLM (logistic regression) analysis to examine if female bias was associated with measures of male proximity. Following the certation hypothesis (Correns 1922), discussed above, we predicted greater female bias in progeny sex ratios for females in which males were in closer proximity. The distance to the closest one to 10 males (*d*_1–10_) was used in these analyses as our measure of male proximity. Distance to the *n*th closest male (*d*_1_–*d*_10_) and population (and their interaction) were fitted sequentially in the model so that the analysis was run for each distance. The best fitting model with the lowest log-likelihood was distance to the second closest male (*d*_2_). We therefore present the distance to the second closest male as the independent variable. Population and the interaction between population and distance were nonsignificant terms and so we subsequently ran a simpler model removing population (see results).

We used heterogeneity *G*-tests (Sokal and Rohlf 1995) using a script written in R to assess if there was significant heterogeneity among maternal families from similar mating environments (high, medium, and low male density) for the two populations, TX-ROS and TX-OAK, with larger sample sizes. For each population, we calculated the pooled *G* (*G*_P_, the pool of all families from a mating environment), heterogeneity *G* (*G*_H_), and total *G* (*G*_T_ = *G*_P_ + *G*_H_) for each mating environment individually (Table 2). This enabled us to examine overall deviations from the expectation of 0.5 (equality) and if there was significant variation in seed sex ratios among mothers from similar mating environments.

**Table 2:** Replicated Goodness-of-fit tests (*G*-test) and the pooled sex ratio for maternal families (SR) in the three mating environments (high, medium, and low male proximity) for two populations of *Rumex hastatulus* (TX-ROS and TX-OAK)

Pop	Mating env.	SR	*df*	*G*_P_	*P*	*df*	*G*_H_	*P*	*df*	*G*_T_	*P*
TX-ROS	High	0.56	1	**6.85**	**0.0089**	7	5.11	0.6460	8	11.96	0.1530
TX-ROS	Medium	0.52	1	0.54	0.4606	11	10.78	0.4619	12	11.32	0.5013
TX-ROS	Low	0.51	1	0.29	0.5930	6	1.89	0.9297	7	2.17	0.9496
TX-OAK	High	0.53	1	1.72	0.1901	7	1.45	0.9841	8	3.16	0.9236
TX-OAK	Medium	0.52	1	0.55	0.4568	6	8.45	0.2072	7	9.00	0.2527
TX-OAK	Low	0.49	1	0.10	0.7506	8	10.22	0.2499	9	10.32	0.3251

*G*_P_ is the pooled *G*-test, *G*_H_ is *G*-heterogeneity test, and *G*_T_ is the Total *G*-test. df is the degrees of freedom for each test, with the total number of families indicated by the df for *G*_T_. Significant *P* values (α = 0.05) are highlighted in bold.

## Results

### Sex-ratio bias and among-population variation in sex ratios

Female-biased sex ratios characterized most populations of *R. hastatulus* (mean sex ratio = 0.62, SE = 0.01, range 0.50 – 0.71; Table 1), with significant female bias in 89% of the populations (41 of 46 populations) that we surveyed. Of the five nonsignificant populations, only two were close to or at equality (sex ratio: OK-WIL = 0.51, AL-BEL = 0.50), whereas the remaining three populations (FL-MIC, NC-KIN, and TX-SUL) had a higher proportion of females (sex ratio = 0.56–0.67), but were small populations with low sample sizes (*n* = 10–66; Table 1). There was no significant difference in the mean sex ratio of populations of the Texas and North Carolina races of *R. hastatulus* (mean TX race = 0.62, SE = 0.01; mean NC race = 0.63, SE = 0.01; GLM race: *F*_1,44_ = 0.45, *P* = 0.505). Comparing all populations from both races, we found significant among-population heterogeneity in sex ratios (*G*_H_ = 293.1, *P* < 0.001; Table 1).

**Figure 1:**
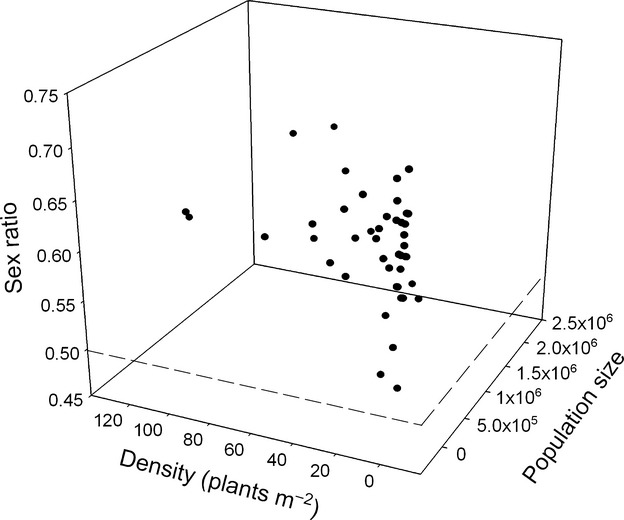
Sex ratio as a function of population size and mean plant density (plants m^−2^) for 45 populations of *Rumex hastatulus*. Sex ratio is represented as the proportion of females (females/females + males). The dashed line represents equal numbers of females and males (sex ratio = 0.5). Overall GLM: *F*_3,41_ = 2.97, *P* = 0.043, GLM Population size: *F*_1,41_ = 0.21, *P* = 0.649, GLM Density: *F*_1,41_ = 0.26, *P* = 0.610, GLM Population size x Density: *F*_1,41_ = 8.44, *P* = 0.006.

Populations of *R. hastatulus* varied considerably in the number of flowering plants (mean number = 224370, SE = 65828, range = 10–≍2,000,000; Table 1) and in plant density (mean density = 21.7, SE = 4.5, range = 0.08–122.4; Table 1). Nonreproductive plants were absent from populations of the Texas race, but were recorded in 19 of the 23 North Carolina race populations (83%) ranging in frequency from 0.1% to 32.0% (Table 1). Individually, population size and density did not explain any of the among-population variation in sex ratios (GLM Population size: *F*_1,41_ = 0.21, *P* = 0.649; GLM Density: *F*_1,41_ = 0.26, *P* = 0.610); however, there was a significant interaction between population size and density (GLM Population size x Density: *F*_1,41_ = 8.44, *P* = 0.006; Fig. 1). Large, high-density populations had the highest proportion of females (greater female-biased sex ratios), whereas small, low-density populations had sex ratios closer to equality (Fig. 1). There was no relation between variation in sex ratios and the percent of nonreproductive individuals for populations from the North Carolina race (GLM % nonreproductive: *F*_1,21_ = 0.50, *P* = 0.487).

### Local density and within-population variation in sex ratio

**Figure 2:**
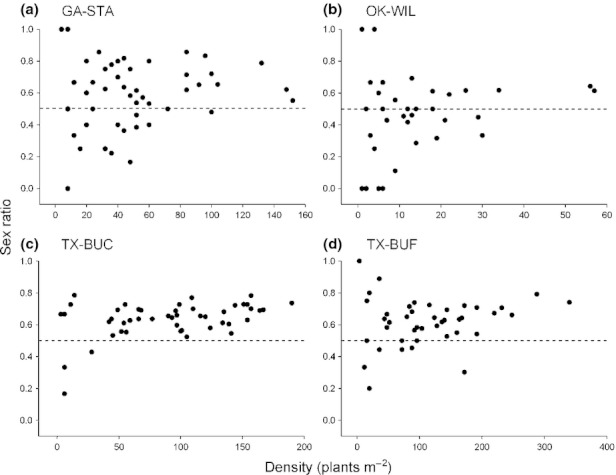
The relation between local sex ratio and density within four populations of *Rumex hastatulus*. Sex ratio is represented as the proportion of females (females/females + males). The dashed line represents equal numbers of females and males.

We investigated whether local plant density was related to sex-ratio variation within 22 populations of *R. hastatulus*. Density had no significant effect on sex ratio in the overall GLMM (GLMM Density: *F*_1,21_ = 0.93, *P* = 0.336), but the effect of the relation varied among populations (GLMM Density × Population: *F*_1,21_ = 2.78, *P* < 0.001). Analysing each population individually, we found a significant relation between local density and sex ratio in four populations (Fig. 2 a–d, Table S1) and a marginally significant relation in another three populations (OK-BAC, TX-GRO, and TX-LIV, GLM Density: *P* = 0.066–0.074; see Table S1). When a significant relation was found, the proportion of females generally increased with plant density and the greatest variation in sex ratio was evident at low density (Fig. 2 a–d).

### Maternal mating environment and progeny sex ratios

**Figure 3:**
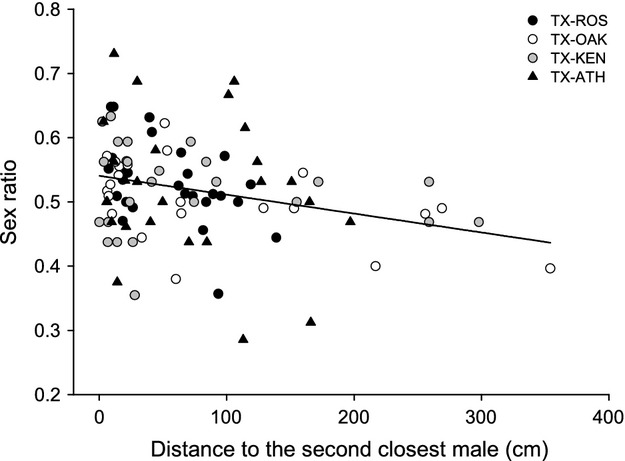
The relation between sex ratio and the distance to the second closest male in four populations of *Rumex hastatulus*. GLM Distance: *F*_1,97_ = 6.84, *P* = 0.01, the equation for this relation is: *y* = *e*^0.1617–0.00105*x*^/(1 + *e*^0.1617–0.00105*x*^).

We found that progeny sex ratios in *R. hastatulus* were significantly related to the distance to the second closest male (Fig. 3, GLM Distance: *F*_1,91_ = 6.13, *P* = 0.015), and this was consistent among the four populations investigated (GLM Population x Distance: *F*_3,91_ = 1.28, *P* = 0.286). There was also no significant difference in overall sex ratio among the four populations (GLM Population: *F*_3,91_ = 0.49, *P* = 0.690). In the simplified model (without population, as it was a nonsignificant term), we found that progeny sex ratios were more female biased when males were in closer proximity to females, compared to where males were more distantly located (Fig. 3, GLM Distance: *F*_1,97_ = 6.84, *P* = 0.01).

We detected no significant heterogeneity in sex ratios among families within each of three mating environments for both TX-ROS and TX-OAK (the two populations with larger sample sizes). Maternal families representing high, medium, and low male density environments had similar sex ratios in both populations (*G*_H_ = 1.45–10.78, *P* > 0.05; Table 2). Pooling families from within each mating environment, we found an increase in the proportion of females with increasing male density (low-to-high male density TX-ROS = 0.51–0.56, TX-OAK = 0.49–0.53; Table 2). There was no significant deviation from equality for the mating environments with low and medium male density in both populations (sex ratio = 0.49–0.52, *G*_P_ = 0.10–0.55, *P* > 0.05; Table 2) and for the high male density environment for TX-OAK (sex ratio = 0.53, *G*_P_ = 1.72, *P* > 0.05; Table 2). In contrast, we found significant female bias in the high male density environment for TX-ROS (sex ratio = 0.56, *G*_P_ = 6.85, *P* < 0.05; Table 2).

## Discussion

Our study of sex-ratio variation in *R. hastatulus* resulted in several major findings: (1) Populations were characterized by female-biased sex ratios, with 89% of the 46 populations sampled containing significantly more female than male plants (average sex ratio 0.62); (2) Variation in sex ratios was associated with population size and plant density; large high-density populations tended to exhibit greater female bias. This pattern was also evident at the patch level within four of the 22 populations, with greater female bias in high-density locations; (3) The maternal mating environment, specifically the proximity of males, had a significant effect on progeny sex ratios in each of the four populations investigated, with more female-biased seed families produced by maternal parents that were closer to males. Below we discuss the significance of these results and evaluate the ecological and genetic mechanisms responsible for governing female-biased sex ratios in *Rumex* species.

### The influence of demographic and genetic factors on sex-ratio variation

Comparative surveys of dioecious flowering plants commonly reveal biased sex ratios, with male-biased ratios approximately twice as common as female-biased ratios (Delph 1999; Barrett et al. 2010; Field et al. 2012a). Understanding the diverse environmental, demographic, and genetic factors causing sex-ratio variation remains a major challenge. An earlier study of sex-ratio variation among 23 populations of *R. hastatulus* from North Carolina reported a small, but consistent female bias (Conn and Blum 1981; Table 1: mean = 0.52, range 0.44–0.60). Our range-wide study revealed a stronger average female bias (0.62) and included five populations sampled from North Carolina, all of which were considerably more female biased (mean 0.65, range 0.63–0.67) than those reported by Conn and Blum (1981). These authors suggested that differences in flowering phenology between the sexes could contribute to seasonal variation in the sex ratio. This is unlikely to be important for our samples of the Texas race of *R. hastatulus,* because all individuals in populations were flowering or fruiting when censuses were conducted. However, nonreproductive plants were observed in 19 of the 23 populations from the North Carolina race, although in the majority (79%) of these populations, they constituted a relatively small proportion (<10%) of the sample. If these nonreproductive plants were largely male, then the sex ratios of North Carolina populations would be closer to the values reported by Conn and Blum (1981). However, we consider this unlikely, as there was no relation between the proportion of nonreproductive individuals and sex-ratio bias in these populations.

We detected a significant interaction between population size and plant density in our analysis of among population variation in sex ratios of *R. hastatulus* (Fig. 1). Large populations with high plant density were significantly more female biased than small populations with sparse plant density. However, neither population size or plant density individually explained a significant component of variation in sex ratios. Similarly, Conn and Blum (1981) reported no effect of plant density on sex ratio in their studies of North Carolina populations. In our study, the effect of local density was not consistent among populations; although in four of 22 populations (18%), sex-ratio variation among patches was associated with local plant density, with greater female bias in higher density patches.

The observed association between demographic factors and sex-ratio variation in *R. hastatulus* raises the question of what mechanism(s) are involved. The patterns we observed are consistent with what is predicted by the certation hypothesis. In small, low-density populations where there are fewer plants and larger distances between the sexes, females may capture limited pollen and as a consequence, seed set may be pollen limited (e.g. Eppley 2005; Steven and Waller 2007; Stehlik et al. 2008). This may be especially important for wind-pollinated species with leptokurtic pollen dispersal curves (Gleaves 1973; Levin and Kerster 1974). The occurrence of sex ratios closer to equality in small, low-density populations of *R. hastatulus* may therefore occur because in these populations, low pollen loads reduce the role that gametophytic competition plays in establishing female-biased seed sex ratios. In contrast, in large populations with high plant densities pollen loads are likely to be considerably greater promoting more intense gametophytic competition and greater female bias. Evidence discussed in the next section on the influence of male proximity on the degree of female bias in seed families supports this hypothesis.

A second and nonmutually exclusive hypothesis that could also contribute to the association we detected between demographic factors and female-biased sex ratios involves sex-specific differences in density-dependent mortality. Under conditions of high-density males may be more susceptible to stress leading to higher rates of mortality. Indeed, a study of the viability of the sexes of *R. nivalis* revealed greater male mortality under more stressful conditions (Stehlik and Barrett 2005) and, in *R. thyrsiflorus* (Rychlewski and Zarzycki 1986) and *R. nivalis* (see Fig. 6 in Stehlik et al. 2007), female bias increases during their perennial life cycles. Interestingly, in *Spinacia oleracea,* another wind-pollinated dioecious annual, the opposite pattern has been reported with females underrepresented in medium- and high-density conditions (Onyekwelu and Harper 1979). Some of the female bias, we observed in very high-density patches of *R. hastatulus* (e.g. >100 plants m^−2^, see Fig. 2) may have resulted from post germination male mortality. However, we may expect these processes to be less important for annual compared to perennial species (Barrett et al. 2010). Indeed, Conn and Blum (1981) found no evidence for sex-differential mortality in a glasshouse study of the influence of density and nutrients on sex ratios of *R. hastatulus*. In our study, density alone had no effect on among-population sex ratios, and within populations, we only detected density effects in four of the 22 populations that we investigated. The inconsistent effects of density on sex ratio could reflect ecological differences among sites affecting the relative survival of males and females. Sex-specific genetic markers, as have been used in *R. nivalis* (Stehlik and Barrett 2005), combined with fine-scale demographic studies, could help assess the extent to which gender-based mortality might influence sex ratios in *R. hastaulus*.

### Local mating environment and progeny sex ratios

The majority of studies of sex-ratio bias in dioecious plants have focused on flowering sex ratios (Barrett et al. 2010), but biased sex ratios can establish at the seed stage and contribute to patterns of sex-ratio variation in natural populations (de Jong and Klinkhamer 2005). In our study, we found that in each of the four populations of *R. hastatulus* that we examined, the local mating environment (male density) influenced progeny sex ratios, with greater female bias where males were in closer proximity. A similar result was also obtained in *R. nivalis* based on a study of male proximity and progeny sex ratios in six populations from the Swiss Alps (Stehlik et al. 2008). These results are best explained by Correns' certation hypothesis (Correns 1922) involving the selective fertilization of ovules by female-determining microgametophytes. Indeed, the strength of gametophytic competition may be particularly important for dioecious taxa with uniovulate flowers, such as *Rumex* species, where pollen tubes compete to fertilize a single ovule. Additional evidence in support of the certation hypothesis comes from experimental studies of the influence of pollen loads on sex ratios in *R. hastatulus* (Conn and Blum 1981; Field et al. 2012b) and other *Rumex* species (Rychlewski and Zarzycki 1975; Stehlik and Barrett 2005). Although in this study, we did not directly measure pollen load size, an association between pollen load and male proximity has been demonstrated in other *Rumex* species (Stehlik and Barrett 2006; Stehlik et al. 2008) and also for experimental arrays of *R. hastatulus* (M. Pickup, D. L. Field and S. C. H. Barrett unpublished data). This suggests that in the present study, and perhaps also for other wind-pollinated dioecious species, variation in male proximity can probably provide a valid surrogate of expected pollen load.

Although male proximity was associated with among-family variation in sex ratios in this study, sex-ratio distorters and X-linked meiotic drive could also contribute to variation in sex ratios (Taylor 1999; Jaenike 2001). These genetic factors are predicted to result in significant among-family variation in progeny sex ratios; however, it remains unclear the extent to which such variation would be influenced in a predictable manner by demographic conditions. After controlling for maternal mating environment (low, medium or high male density), we found no significant heterogeneity in sex ratios among maternal families, a result that would appear to be inconsistent with sex ratio distorters (or restorers) playing an important role in this system. Larger family sizes could provide greater power to identify families in which genetic modifiers of the sex ratio occur, but at the present time, there is no conclusive evidence for their occurrence in *Rumex* species or in the vast majority of other dioecious plants.

We have assumed that the flowering sex ratios that we measured from open-pollinated families of *R. hastatulus* in the glasshouse approximate seed sex ratios. However, without sex-specific genetic markers to determine seed sex ratios (e.g. Stehlik and Barrett 2005), we cannot preclude the possibility that female and male seeds might differ in viability. In our study, germination rates were generally high (range of mean values for the four populations = 80.6–96.5%), and an earlier study of *R. nivalis* using sex-specific markers found very similar sex ratios in seed and seedlings, indicating no difference in viability between female and male seeds (Stehlik and Barrett 2005). Moreover, a study of open-pollinated progeny sex ratios in populations of *R. nivalis*, specifically comparing the methods we used here with sex-specific markers, found no difference in sex ratios (Stehlik et al. 2008). After germination, there was very little mortality of plants with survival to flowering >97% in all populations, thus the scope for bias to be introduced in our glasshouse study seems likely to have been quite minimal.

## Conclusions

Deviations from equal sex ratios in populations of dioecious plants result from diverse ecological and genetic factors. Our results on the influence of population size, plant density, and the composition of the local mating neighborhood in *R. hastatulus* highlight the interaction of ecology, demography, and genetics for sex-ratio variation. Although the importance of certation has been controversial (Carroll and Mulcahy 1993; Taylor et al. 1999), our study provides further evidence in support of the hypothesis that this mechanism contributes toward female-biased sex ratios.

The contribution of gametophytic competition to sex-ratio bias may be greater for annual species, as there are fewer opportunities for sex-specific differences in mortality to accumulate during their relatively brief life cycles, in comparison with longer lived perennial species. Interestingly, the average degree of bias (0.62) that we found for *R. hastatulus* is significantly lower than has been reported for perennial dioecious *Rumex* species (e.g. *R. acetosa* – mean 0.79, Korpelainen 2002; *R. nivalis* – mean 0.82, Stehlik and Barrett 2005). However, we also found less biased seed sex ratios in *R. hastatulus* (mean = 0.52) compared to *R. nivalis* (mean = 0.62; Stehlik et al. 2008), despite much higher overall plant densities in *R. hastatulus* and probably larger expected pollen loads. The difference in seed sex ratios may reflect both life history and demographic differences between the species. For example, the annual life cycle and larger effective population sizes in *R. hastatulus* may increase the efficiency of selection and reduce the accumulation of deleterious recessive alleles on nonrecombining regions of the Y chromosome (Charlesworth and Charlesworth 2000; Charlesworth 2002). This might conceivably lead to a reduced intensity of gametophytic competition under higher pollination intensities. Future comparative studies of sex ratios in *Rumex* species with contrasting life histories and demography could clarify the extent to which ecological factors interacting with the genetic systems of species contribute toward sex-ratio variation across the genus.

## Supporting Information

**Table S1**. Results of the Generalized Linear Mixed Model (GLMM, logistic regression) analyses to examine the relation between local density and sex ratio within 22 populations of *Rumex hastatulus*. Populations with significant relationships are highlighted in bold and results indicated with the level of significance (***, *P* < 0.001; ** *P* < 0.01; *, *P* < 0.05; †, 0.05 < *P* < 0.1). Transect and quadrat (within transect) was fitted to the random model. *n* is the number of quadrats in each population.
