# Supplementary material for: The influence of demography and local mating environment on sex ratios in a wind-pollinated dioecious plant
Source: Ecol Evol. 2013 Feb 6;3(3):629–39. doi: 10.1002/ece3.465 (PMC3605851; doi:10.1002/ece3.465)
Supplement: Supplementary file 1 [file ece30003-0629-SD1.docx]

**Supporting information**

Table S1. Results of the Generalised Linear Mixed Model (GLMM, logistic regression) analyses to examine the relation between local density and sex ratio within 22 populations of *Rumex hastatulus*. Populations with significant relationships are highlighted in bold and results indicated with the level of significance (***, *P* < 0.001; ** *P* < 0.01; *, *P* < 0.05; †, 0.05 < *P* < 0.1). Transect and quadrat (within transect) was fitted to the random model. *n* is the number of quadrats in each population.

| **Population** | **Fixed factor** | ***n*** | ***F*** | ***P*** |
| --- | --- | --- | --- | --- |
| AL-BRU | Density | 41 | 0.98 | 0.328 |
| FL-CHI | Density | 57 | 0.18 | 0.670 |
| FL-GAI | Density | 42 | 2.59 | 0.115 |
| GA-ELL | Density | 30 | 0.02 | 0.887 |
| **GA-STA** | **Density** | **66** | **5.81** | **0.019^*^** |
| LA-MAN | Density | 54 | 0.42 | 0.517 |
| NC-BAT | Density | 45 | 0.13 | 0.724 |
| NC-HIC | Density | 40 | 0.72 | 0.400 |
| NC-ROS | Density | 37 | 0.28 | 0.598 |
| **OK-BAC** | **Density** | **51** | **3.33** | **0.074^†^** |
| **OK-WIL** | **Density** | **37** | **7.62** | **0.009^**^** |
| SC-MAR | Density | 37 | 2.22 | 0.145 |
| TX-ATH | Density | 70 | 1.65 | 0.204 |
| **TX-BUC** | **Density** | **45** | **8.59** | **0.005^**^** |
| **TX-BUF** | **Density** | **41** | **8.41** | **0.006^**^** |
| **TX-GRO** | **Density** | **25** | **3.5** | **0.073^†^** |
| TX-KEN | Density | 51 | 0.12 | 0.730 |
| **TX-LIV** | **Density** | **73** | **3.48** | **0.066^†^** |
| TX-MTP | Density | 61 | 0.01 | 0.914 |
| TX-OAK | Density | 49 | 0.32 | 0.574 |
| TX-ROS | Density | 74 | 2.73 | 0.102 |
| TX-WES | Density | 72 | 0.82 | 0.368 |
